# Biology and clinical relevance of EpCAM

**DOI:** 10.15698/cst2019.06.188

**Published:** 2019-05-21

**Authors:** Laura Keller, Stefan Werner, Klaus Pantel

**Affiliations:** 1 Institute of Tumor Biology, University Medical Center Hamburg-Eppendorf, Hamburg, Germany.

**Keywords:** EpCAM, liquid biopsy, circulating tumor cells, EMT, Tumor biomarker, tumor cell dissemination

## Abstract

Epithelial cell adhesion molecule (EpCAM) is a transmembrane glycoprotein primarily known to mediate homotypic cell contacts in epithelia tissues. Because EpCAM expression is limited to normal and malignant epithelia, it has been used as diagnostic marker for the detection of carcinoma cells in mesenchymal organs such as blood, bone marrow or lymph nodes. In particular, the detection and molecular characterization of EpCAM-positive circulating tumor cells (CTCs) in the blood of carcinoma patients has gained considerable interest over the past ten years. EpCAM is primarily considered as an adhesion molecule, but recent studies have shown diverse biological functions including regulation of cell proliferation and cancer stemness. In this review, we summarize the current knowledge on the biological properties of EpCAM with emphasis on mechanisms involved in cancer progression and discuss the clinical implications of these findings for the clinical use of EpCAM as a diagnostic marker.

## INTRODUCTION

The EpCAM protein was discovered almost 40 years ago as a major epithelial carcinoma antigen by M. Herlyn and colleagues, as a result of its property to generate monoclonal antibodies binding specifically to human colorectal carcinoma cells [1]. In the following, the protein has been independently described many times as a highly immunogenic tumor-associated antigen. In these studies, the discovered antigen received the name of the respective monoclonal antibody recognizing it (a summary table of its different names can be found in [2] and [3]). Specifications of the identified antigens and subsequent cloning of the corresponding genes, in each case, lead to their identity as EpCAM [4]. Since 2007, the nomenclature has been harmonized and it has been agreed that the protein as well as its encoding gene (*EPCAM*) shall be called EpCAM, which is the abbreviation for Epithelial Cell Adhesion Molecule due to the first reports from Litvinov and colleagues on its adherent function in epithelial cells [4].

Liquid biopsy in oncology has gained increasing interest during the last decade revealing potential to change clinical practice by exploiting peripheral blood as a source of information about tumor status and treatment options [5]. Liquid biopsy is a general denomination introduced by Pantel and Alix-Panabieres approximately ten years ago [6], which refers to any tumorderived analyte present in body fluids like peripheral blood, urine, bone marrow and salvia. Nevertheless, of peculiar interest is the detection of circulating tumor cells (CTCs) and tumor-derived soluble molecules or particles, such as circulating tumor DNA (ctDNA) and other circulating nucleic acids (in particular microRNAs), extracellular vesicles and tumor-educated platelets in the blood circulation of cancer patients. The greatest challenge of this field is to specifically discriminate the tumor-derived analyte from a tremendously high background composed of healthy cells and their content. The majority of publications on liquid biopsy focuses on CTCs that allow to obtain a broad range of information at the DNA, RNA and protein level [7-10]. As the vast majority of cancers are of epithelial origin, targeting epithelial antigens was the first approach to discriminate a tumor cell among millions of peripheral blood mononuclear cells that are of mesenchymal origin. EpCAM therefore became the most commonly used epithelial marker for the capture of CTCs in the blood circulation of carcinoma patients.

Here, we will review the accumulating recent evidence that EpCAM is a special tumor marker with profound biological properties far beyond inter-cellular adhesion. This knowledge will open new avenues for the use of EpCAM as a diagnostic liquid biopsy marker in cancer patients.

## BIOLOGICAL PROPERTIES OF EpCAM

### *EPCAM* gene mutations

The *EPCAM* gene consists of 14 kb in total and is located on chromosome 2 (2p21). The gene is conserved across many different species from zebrafish to humans. Particularly the amino acid (aa) sequence of the extracellular domain is conserved to a high extent from fishes to primates, suggesting the functional importance of the EpCAM protein [11]. Mutations in the EPCAM gene have been identified in two hereditary syndromes. In congenital tufting enteropathy (CTE), a rare autosomal recessive form of intractable diarrhea of infancy and Lynch Syndrome also known as Hereditary Non-Polyposis Colorectal Cancer (HNPCC), which is one of the most common cancer susceptibility syndromes that predisposes to colorectal adenocarcinoma, endometrial carcinoma, and various other cancers. In CTE, biallelic EPCAM mutations are mostly loss of functions mutations, predicted to affect EpCAM protein structure, disrupting its expression and/or stability [12]. Constitutive and inducible CTE-associated murine models have been developed by engineering EPCAM KO mice. These models show enhanced intestinal permeability and migration as well as decreased ion transport. The consequences of EpCAM loss in this disease are complex, including decreased expression of tight junctional proteins like Claudins [13, 14] or dysregulation of E Cadherin and ß Catenin leading to disorganized transition from crypt to villi [15]. Lynch syndrome is caused by inheritance of one defective allele in genes involved in DNA mismatch repair (MMR) machinery, predominantly MSH2, MLH1, MS2 and MSH6. Contrary to CTE, EPCAM-associated Lynch syndrome is not due to loss of EpCAM per se, but rather is due to monoallelic deletions of the 3′ end of the *EPCAM* gene in which the polyadenylation signal is lost leading to *MSH2* promoter hypermethylation, read-through transcription of the *EPCAM* and *MSH2* genes, and loss of MSH2 protein expression [16].

### EpCAM protein structure

**Figure 1:**
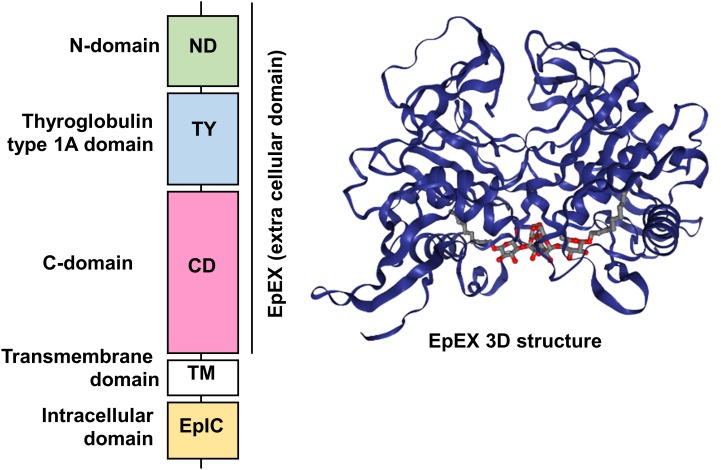
FIGURE 1: Schematic diagram of the domain structure of full length EpCAM protein and crystal structure of an extracellular EpCAM *cis*-homodimer according to Pavsic *et al*. Full length EpCAM consists of a N-terminal signal peptide (SP) followed by three compactly folded extracellular domains (N-Domain (ND), Thyroglobulin type 1A domain (TY) C-Domain CD), a single spanning transmembrane domain (TM) and a c-terminal intracellular domain (EpIC). Two EpCAM subunits form a heart-shaped dimer on the cell surface [11]. Protein Data Base entry 4MZV.

Human EpCAM protein is a transmembrane glycoprotein polypeptide of 314 aa, consisting of a large N-terminal extracellular domain (EpEX) of 242 aa and 27 kDa, a single-spanning transmembrane domain (TM) of 23 aa and 2 kDa and a short C-terminal cytoplasmic domain of 26 aa and 3 kDa (EpIC; **Figure 1**).

Significant insights in the structure of the EpCAM protein were recently gained thanks to the crystallization of a non-glycosylated form of the EpEX domain by Pavsic and colleagues that is lacking the N-terminal signal peptide [11]. The authors found out that the extracellular part of human EpCAM forms a heart-shaped dimer, which would form at cell surfaces. The polypeptide chain of EpEX is folded into a compact shape made of three domains (N-Domain ND, Thyroglobulin type 1A domain TY, and C-Domain CD) arranged in a triangular fashion where each domain contacts the other two. The extracellular domain also presents three N-glycosylation sites (Asn74, 111, 198), implicated in protein stability and covering the lateral protein surfaces (**Figure 1**) [11].

One function of the intracellular cytoplasmic domain is to anchor the EpCAM protein to the cytoskeleton, as demonstrated by Balzar and colleagues via an interaction with α-actinin [17]. At the C-terminus, amino acids 312-314 display a putative PDZ binding site, which has been shown in other intercellular contact proteins to be key in complex formation with signaling or structural proteins [2]. In line with this, a short segment of the cytoplasmic tail was found to resemble the inhibitory domains of PKCs and could cause PKC inhibition [18]. *In vivo*, EpCAM forms a *cis*-dimer of two EpCAM molecules on the surface of the same cell that approximately protrude 5 nm form the cell surface. The dimerization depends on the loop of the TY domain (involved in interactions with the CD of the other molecule) and also on the transmembrane helix [11]. We will discuss in the following chapter how these structural insights can help to refine the different cellular roles of EpCAM.

The EpCAM protein contains several cleavage sites that are essential for its biological activity as well as for controlling protein expression. It is worth mentioning that soon after identification of the EpCAM protein, a cleavage at position Arg-80/Arg-81 of the TY loop was discovered. Cleavage of EpCAM at this position results in a 6 kDa N-terminal fragment that remains bound to the protein backbone by the first disulfide bond within the TY-like domain and importantly, would lead to the disruption of the *cis*-dimer as experimentally demonstrated on the EpEX domain [11]. *In vivo*, this cleavage is supposed not to be frequent (see below).

Regulated Intramembrane Proteolysis (RIP) was initially identified as a new form of membrane-to-nucleus signaling mechanism. Instead of propagating signals through a cascade of intermediate messengers, transmembrane receptors directly respond to stimuli by undergoing RIP. RIP describes an evolutionary conserved mechanism that consists in the cleavage of transmembrane proteins within the plane of the membrane to liberate biologically active cytosolic fragments that enter the nucleus to control gene transcription [19, 20]. Another consequence of this mechanism would be also the degradation of the protein substrate [21]. RIP proceeds in two essential steps. First, the extracytosolic (luminal or extracellular) domain is removed by the action of sheddases, principally ADAM 10 and 17 at α sites. Then, the secondary cleavage requires the inter- vention of multiprotein complexes like γ-secretase, able to hydrolyze proteins in the hydrophobic environment of the membrane bilayer [19].

**Figure 2:**
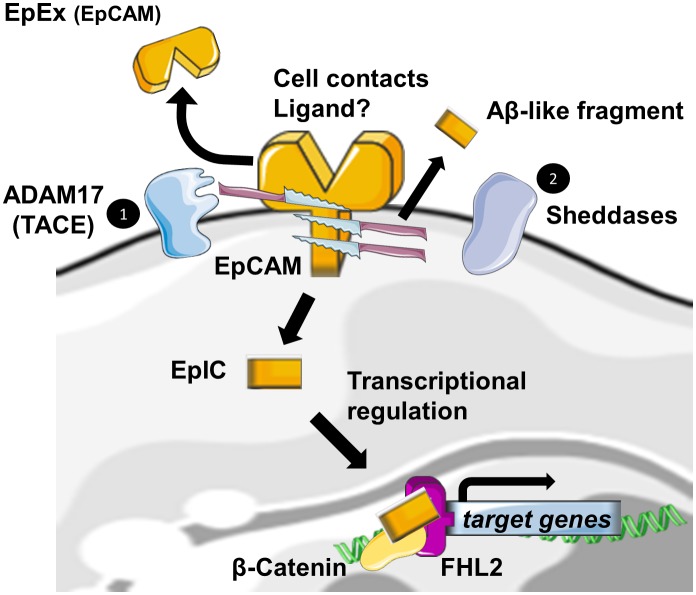
FIGURE 2: EpCAM cleavage and downstream signalization. Cellular contacts, binding of EpEX or another unknown lig- and, lead to the activation of EpCAM cleavage by disintegrin and metalloprotease (ADAM17), and the subsequent release of the soluble EpEx domain in the intercellular space. In a second step, sheddases are acting at several sites in the transmembrane domain and generate Aβ-like fragments and an intracellular domain EpIC. If EpIC has been shown as a signalisation molecule, the functions of Aβ-like fragments are still unknown.

Also, EpCAM has been identified, among many other transmembrane receptors, to undergo RIP [22]. The first cleavage, mediated by ADAM17, which is also called TACE for TNFα Converting Enzyme, triggers the release of the soluble fragment EpEX in the surrounding environment where it could act in a juxtacrine manner as a homophilic ligand for non-cleaved EpCAM [23]. Note that cell-to-cell contact is also another initial trigger for RIP of EpCAM [23]. The second cleavage by γ-secretase complexes occurs at two distinct ε- and γ-sites and respectively lead to soluble extracellular Aβ-like fragments and intracellular domain EpIC release in the cytosol of the cell. Tsaktanis *et al*. have recently identified by mass spectrometry the precise position of the cleavage sites of human EpCAM [24]. Although? the functions of the Aβ-like fragment are still unknown, EpIC plays a central role in downstream signalization of EpCAM (**Figure 2**).

According to the extracellular domain structure, ε- and γ -sites are always exposed in the dimeric state of the protein, whereas α sites involved in initiation of RIP and Arg80-Arg81 do not seem to be easily accessible to proteases. They are indeed directly involved in *cis*-dimerization interactions or sterically hindered by the glycan chains attached to Asn74 and Asn111. As only a fraction of total EpCAM is cleaved, Pavsic *et al*. suggested the existence of a dynamic equilibrium between monomeric and dimeric EpCAM, conformational changes induced by external yet unknown ligands of EpCAM or by sheddases themselves. The monomer–dimer equilibrium could be affected by various factors, such as lipid composition or association with other proteins [11]. BACE1 is a sheddase that has already been identified in EpCAM cleavage. Because of its optimum at pH 4.5 for enzymatic activity, BACE1 is functional in acidic intracellular compartments, including the trans-Golgi network and endosomes [25]. As full exposure of the cleavage site in the protein is achieved with the destabilization of the EpCAM dimer by a pH drop, shedding through BACE1 was suggestive of an internalization of EpCAM into acidic intracellular compartments through endocytosis. Tsaktanis *et al*. demonstrated this phenomenon and its dependence on clathrin proteins [24].

### Regulation of EpCAM expression

*EPCAM* gene expression is controlled on the transcriptional level. The proximal promoter region of human *EPCAM* that predominantly controls gene transcription specifically mostly in epithelial tissues has been cloned and many transcriptions factors binding sites within this sequence have been reported so far [26]. The sequence upstream of the transcription start site (TSS) has been defined and *in silico* analysis of the EpCAM promoter revealed the lack of typical TATA and CAAT boxes but the presence of eukaryotic promoter elements such as initiator consensus sequences and GC boxes, as well as consensus binding sequences for transcription factors like SP-1, activator protein 1 (AP-1), activating protein 2 (AP2), Ets, ESE-1 and E-pal-like transcription factors, which are known to play a role in epithelial specific expression [26]. However, little biological data supports an actual role for these transcription factors in *EPCAM* gene expression. In metastatic lymph nodes from lung, breast and pancreas cancers, the upregulation of Ets family transcription factor Esx/Elf3 in metastatic lymph nodes correlated well with expression of EpCAM [4]. In ovarian cancer, Van der Gunt *et al*. confirmed binding of several transcription factors (AP2*α*, Ets1, Ets2, E2F2, E2F4 and STAT3) within the *EPCAM* gene by chromatin immunoprecipitation [27]. Moreover, also the tumor suppressor gene p53 was identified as a repressor of EpCAM expression and by chromatin immunoprecipitation assay, the binding of wild type p53 to a site located within intron 4 was confirmed [28]. Lastly, transcription of *EPCAM* was shown to be activated by TCF/β-catenin pathway via the identification of two TCF binding elements in the *EPCAM* promoter that specifically bound to TCF-4 [29]. Since the intracellular domain of the EpCAM protein (EpIC) can directly interact with the TCF/β-catenin protein complex, this may create a positive-feedback loop on EpCAM expression at the level of gene transcription [22], which still needs to be proven on the experimental level.

So far, few microRNAs controlling *EPCAM* mRNA expression have been identified. MicroRNA-181 has been shown to upregulate *EPCAM* gene expression, possibly via a positive feedback loop between miR-181 and Wnt/*β*-catenin signaling [30]. In prostate cancer, miRNA200c and miRNA205 were shown to induce expression of *EPCAM* mRNA and protein [31]. However, whether it is a direct or indirect mechanism is not known. Nevertheless, to better understand and monitor tumor cell dissemination, the identification of transcription factors or of microRNAs that govern *EPCAM* gene expression and that are implied in Epithelial-Mesenchymal Transition (EMT) is of high interest in the context of tumor diagnosis, as outlined below. 

Already in 1994, it was described that *EPCAM* gene expression is also controlled on the epigenetic level. It was shown that DNA methylation could prevent amplification of a transfected *EPCAM* gene and this mechanism was suggested to occur in tumor cells [32]. Interestingly, it was confirmed almost ten years later that mutations of TP53 induce loss of DNA-methylation and amplification of the *EPCAM* gene [33]. However, whether DNA methylation of *EPCAM* gene influences DNA amplification via a replication or recombination dependent mechanism has not been identified. Together with the results that loss of p53 could also enhance EpCAM expression at the transcriptional level, these results could provide interesting mechanistic clues to understand EpCAM overexpression in cancer.

On the other hand, DNA methylation that occurs mainly on CpG islands of the promoters of the genes generally lead to their transcriptional silencing. Some studies have since then investigated the methylation of the promoter in various cancer types. In breast cancer, DNA hypomethylation did not correlate to tissue expression [34]. In the contrary, in ovarian, oral squamous cell carcinoma, colon and lung cancers, expression of EpCAM was correlated with DNA methylation in tissues from cancer patients. For a more complete review on DNA methylation regulation of EpCAM, see [35].

Two studies reported on enzymes and histone modifications involved in epigenetic regulation of the *EPCAM* gene. Chromatin immunoprecipitation revealed an association of repressive epigenetic marks and methylation within the *EPCAM* promoter increased gradually as *EPCAM* expression decreased in three lung adenocarcinoma cell lines [36], whereas in ovarian cancer positive cell lines, epigenetic marks that indicate activated gene transcription were immunoprecipitated with *EPCAM* promoter sequences [37].

The histone acetyl transferase p300/CBP was furthermore shown to contribute to repression of *EPCAM* gene expression in response to TNFα stimulation. Mechanistically, TNFα stimulation led to the activation of the transcription factor NF-kB, which then recruits p300/CBP and thereby could compete for this limited pool of cotransactivators [38].

### EpCAM expression in healthy tissues

Tissue distribution of EpCAM has widely been investigated by immunohistochemical staining [39, 40]. A strong positive signal, mainly concentrated to lateral and basal membranes, was obtained for most epithelial cell types throughout the body but not in any non-epithelial tissue like lymphoid origin and bone marrow-derived cells, mesenchymal, muscular or neuroendocrine tissue. Expression levels of EpCAM vary between different organs and cell types. In adults, epithelia of the colon, small intestine, pancreas, liver, gall bladder and endometrium owns the highest expression [39]. In general, EpCAM expression is positively correlated with proliferative and negatively correlated with more differentiated areas. One example is the epithelium of the intestine of the rat, in which a decreasing EpCAM gradient can be observed from crypts to villi, corresponding to high EpCAM expression in the intestinal stem cells which are located in the crypts and decreasing levels in the differentiated cells at the top of the villi [41]. Progenitor cells of skin epithelium express EpCAM, whereas differentiated keratinocytes do not [37]. In liver, EpCAM expression has also been observed in the precursor stem cells during regeneration processes, sustaining its role as an epithelial stem cells marker.

### EpCAM expression in cancer

In the majority of cancer tissues, EpCAM is frequently overexpressed [39]. In contrast, the majority of squamous cell carcinomas show lower EpCAM expression than adenocarcinomas and EpCAM was found to be absent in sarcomas, lymphomas, melanomas, and neurogenic tumors [39, 40]. Especially high abundant levels of EpCAM expression can be observed in carcinomas derived from colon, intestine, breast, lung and prostate. Contrary to healthy epithelia, the distribution of EpCAM varies depending on the type of carcinoma, from a basolateral to a homogenous whole cell membranous distribution. Additionally, strong EpCAM signals can also be detected in the cytoplasm and nuclei, since EpCAM is subject to regulated proteolytic cleavage [39].

The prognostic value of EpCAM expression is dependent on the cancer type. In some carcinoma types (thyroid, renal clear cell, head and neck squamous cell carcinomas), EpCAM immunostaining has been associated with improved survival [42, 43], whereas in other carcinoma types like pancreas, bladder, gall bladder, gastric, NasoPharynx Carcinoma, EpCAM expression is associated with decreased survival [44, 45]. Interestingly, for colorectal, ovarian, lung and breast carcinomas both roles have been reported [27, 43]. Thus, it seems that impact of EpCAM expression is context dependent. In breast cancer for instance, EpCAM is associated with an unfavorable prognosis in the luminal and basal-like intrinsic subtypes but with a favorable prognosis in the HER2 intrinsic subtype [46].

**Figure 3:**
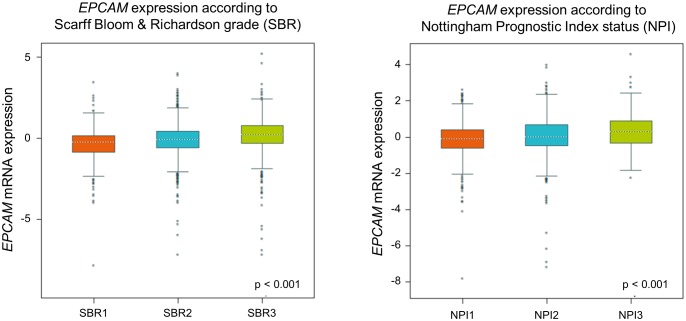
FIGURE 3: Targeted expression analysis of normalized gene expression values of *EPCAM* in groups of Scarff Bloom & Richardson grade status (SBR) and Nottingham Prognostic Index status (NPI) using the Breast Cancer Gene-Expression Miner v4.1 (bc-GenExMiner v4.1).

To re-evaluate clinical relevance of *EPCAM* gene expression we utilized in a large published breast cancer cohort by using the Breast Cancer Gene-Expression Miner v4.1 [47]. In this analysis, we found a gradual rise of ECPAM gene expression related to increasing tumor grades and prognostic index status (**Figure 3**), indicating an increase in EPCAM expression during breast cancer progression. For more detailed reviews on the prognostic value of EpCAM expression in cancer, see [43].

Nevertheless, an important detail is that most of these studies do not distinguish between expression and localization of the extracellular and intracellular EpCAM domains. Given the different roles of these domains, this distinction could be useful to better understand the role of EpCAM in tumorigenesis. In a retrospective study comparing oral squamous cell carcinoma, oral dysplasia and normal tissue, immunohistochemical analysis of nuclear and cytoplasmic Ep-ICD and EpEx was correlated with worse disease outcome for oral dysplasia patients [48]. In thyroid carcinomas, nuclear Ep-ICD accumulation predicted poor prognosis and was elevated in patients with anaplastic tumors [49]. In a retrospective study on breast cancer, tissues were analyzed by immunohistochemistry to determine the expression patterns of nuclear and cytoplasmic Ep-ICD and membranous EpEx and correlated with clinicopathological parameters and follow up. Nuclear Ep-ICD was identified as the most significant predictive factor for reduced disease-free survival in patients suffering from invasive ductal carcinoma. The high recurrence of disease in nuclear Ep-ICD positive patients, especially those with early tumor stage suggests that nuclear Ep-ICD accumulation holds the promise of identifying early stage patients with aggressive disease who are likely to need more rigorous post-operative surveillance and/or treatment [50]. Munz *et al*. have also discovered that differential glycosylation patterns of the EpCAM protein can discriminate normal from malignant tissues. EpCAM was shown to be hyperglycosylated in carcinoma tissue as compared with autologous normal epithelia. Interestingly, glycosylation of EpCAM at asparagine198 was shown to be crucial for protein stability as shown by mutagenesis of EpCAM substitution of asparagine198 for alanine led to decreased overall expression and half-life of the molecule at the plasma membrane, which is of considerable importance with respect to EpCAM variants expressed in normal and cancer tissue [51].

### Cancer-related functions of EpCAM

#### Role in intercellular adhesion

Adhesive interactions of cells play an important role in the establishment and maintenance of tissue architecture. Based on their characteristic domain structure, the majority of cell surface adhesion molecules can be grouped into four families: cadherins, integrins, selectins, and cell adhesion molecules (CAMs) of the immunoglobulin (Ig) super-family. EpCAM does not belong to either of the four major families of CAMs, but was first established to mediate Ca^2+^-independent homophilic intercellular adhesions when introduced in cells that lack their own means of cell-cell interaction [52, 53]. EpCAM was not associated with any classical junctional structures but would promote cell-cell contact via the formation of intercellular *trans*-oligomers [54, 55]. On the molecular level EpCAM interacts with different adhesion proteins like CD44, Claudins and E-Cadherin [56].

Inconstant results, i.e. the cleavage or knockout (KO) of EpCAM does not affect cell adhesion [24], reported since then, recently led Lenarcic and colleagues to reinvestigate its cell adhesion property and its oligomerization capability via various analytical techniques [57]. Their data demonstrate that both EpCAM and EpEX could form *cis*-dimers *in vitro* and *in vivo*, but no notable higher-order oligomerization. Moreover, EpCAM molecules from adjacent cells do not form inter-cellular higher-order homo-oligomers, suggesting for the authors that EpCAM's function as a homo- philic CAM was highly implausible [57]. Nevertheless, even without direct involvement in formation of cell-cell contacts, EpCAM was initially considered to function as cell-cell adhesion protein thanks to its intracellular domain interaction with the actin cytoskeleton via α-actinin [58]. It also interacts with several important CAMs and regulates adhesive structures between cells and cell-matrix, including TJs, AJs, desmosomes, and hemi-desmosomes.

It was shown that EpCAM can modulate the strength of cellular adhesion mediated by E-Cadherin by disrupting the link between α-catenin and F-actin [59]. However, another study reports opposite results by showing an increase in total cellular α-catenin following EpCAM down-regulation, leading to an improved anchorage of the E-Cadherin/α-catenin/β-catenin complex to the cytoskeleton [60]. EpCAM also modifies tight junctions'composition and functions by regulating amounts and locations of claudins via a direct interaction of its transmembrane domain with claudin-7 [61, 62]. These results have not been fully confirmed in viable *EpCAM* KO animal models yet. While a study by Lei *et al*. found dysfunctional tight junctions in a CTE mouse model with EpCAM inactivation [63], in a study by Guerra *et al*. a similar mouse model of CTE points out that adhesion junctions and E-Cadherin/beta-Catenin are affected by EpCAM loss [15]. For an exhaustive review of EpCAM's role in adhesive structures, see [64].

Interestingly, association of EpCAM, E Cadherin and integrin α*v*β*6* on tumor cells can also play additional role and trigger tumor-mediated fibroblast activation, thereby influencing both gene expression and tumor response to therapeutic agents [65].

#### Role of EpCAM in cell proliferation

The first hypothesis that EpCAM can play a role in regulating cell proliferation came from afore mentioned observation in healthy tissues that EpCAM is preferentially localized to proliferative areas [3]. Additional evidence for an active role of EpCAM in regulation of cell proliferation came from the positive correlation between EpCAM expression and cell proliferation observed in epithelial and fibroblastic cell lines [66]. Treatment of human colon and lung cancer cell lines with the specific EpCAM antibody G8.8 showed a dose-dependent increase in proliferation and revealed that most deregulated genes were involved in cell cycle regulation (like *LATS2, FOSL2* and *PIM1*), proliferation, cell growth, apoptosis (mainly *GADD45* and *PIM1*) and other cancer related processes [67]. EpCAM siRNA treatment resulted in a significant decrease in cell proliferation in breast cancer cell lines [60]. Tumors expressing EpCAM implanted in immunodeficient mice were furthermore strongly positive for the proliferation marker Ki67 [22]. Moreover, expression levels of EpCAM correlate with de-differentiation and malignant proliferation of epithelial cells. In 1996, Litvinov already noticed that both the level of EpCAM expression and the number of positive cells increased with the grade of carcinogenesis in cervical intraepithelial neoplasia [68]. In patients suffering from gastric cancer, high EpCAM expression has also been linked to proliferation, assessed by Ki67 staining [44].

Mechanistically, EpIC the soluble intracellular domain of EpCAM constitutes the signaling active intracellular compound. It is found in a large nuclear complex together with FHL2, β-catenin and Lef-1. Importantly, in this complex, FHL2 might act as a scaffold protein and links EpCAM to the Wnt pathway via interactions with β-catenin and Lef-1. This nuclear complex then binds promoters of genes involved in cell cycle regulation like c-MYC, cyclin A and E [69]. It is still unclear whether EpCAM can directly activate components of the cell cycle machinery or if EpCAM-mediated proliferation is a secondary effect of e.g. MYC upregulation, repression of apoptosis, elevation of cell metabolism or interruption of anti-proliferative signals [70]. In 2013, also Chavez-Perez *et al*. showed that EpCAM controls cell cycle progression via the regulation of the key player cyclin D1 at the transcriptional level [70]. There is also more recent evidence that the soluble EpEx can sustain cell proliferation by acting as a ligand of EGFR in head and neck squamous cell carcinomas and colorectal cancers by inducing cell signalization through ERK1/2 [42, 71] and AKT [71].

#### Role of EpCAM in stemness

EpCAM is mostly expressed in epithelial cells but likewise also expressed in various tissue stem cells, precursors, and in murine and human embryonic stem cells [69, 72], which has important implications for cancer progression. EpCAM expression is tightly regulated at earliest time points of gastrulation in order to achieve a mandatory spatiotemporal cellular heterogeneity of EpCAM in endo-and mesodermal lineages [73]. It has also been associated with morphogenesis based on the marked variations of its expression during development and regeneration of epithelia. In later stages of epithelial development, EpCAM acquires a strictly epithelial-specific expression, whereas in terminally differentiated cells EpCAM is not expressed [3, 74, 75]. For example, in liver, EpCAM was only detected in regenerating cells like hepatobiliary stem and progenitor cells, while it was lost in mature hepatocytes.

Key signals implicated in stem cell phenotype are provided by components of the Wnt pathway, LIF/STAT3 and c-Myc, and by the transcription factors Nanog, Oct3/4, Klf4, and Sox2. Indeed, these factors play a central role to the conversion of somatic cells into induced pluripotent stem cells [76]. Mechanistically, EpIC and the Wnt pathway are linked to each other, notably via FHL2 scaffold protein (see above). Moreover, they have also been demonstrated to be direct targets of EpCAM in human embryonic stem cells [77]. Recently, Kuan *et al*. suggested that EpEX and EpCAM could also trigger reprogramming of fibroblasts into induced pluripotent stem cells via activation of STAT3 [78].

Cancer stem cells share many similar biological properties with embryonic stem cells. Precisely, cancer stem cells, like embryonic stem cells, undergo molecular regulations such as persistent self-renewal. EpCAM has been used in combination with CD44 as a marker to efficiently isolate cancer stem cells in different cancer entities such as colon, breast, pancreas and prostate carcinomas [79]. Especially in tumor-initiating cells from the liver, an important function for the EpCAM protein has been well described [29]: an interrelation of EpCAM and Wnt in hepatocellular carcinomas was sustained with the finding that the *EPCAM* gene becomes transcriptionally activated by TCF-4, a member of the Lef family of transcription factors [29]. Taken together, these findings depict an important role for EpCAM in the induction and/or maintenance of proliferation and cellular differentiation of progenitors, stem cells, induced pluripotent stem cells, cancer cells, and cancer stem cells. Stem cell phenotypes and mesenchymal characters have often been conflated [80, 81]. Interestingly, some studies also report a parallel regulation of reprogramming factors and EMT-TF by EpCAM [45, 82].

#### EpCAM and Epithelial to Mesenchymal Transition (EMT)

EMT is nowadays described as a complex program, governed by specific transcription factors, miRNA, epigenetic and post-translational regulators and executed in response to pleiotropic factors that leads to the modification of the adhesion molecules expressed by the cell and the further acquisition of a migratory and invasive behavior [83]. Contrary to E Cadherin, biological regulation of EpCAM during EMT seems to be less well established. Therefore, in the following paragraph, we will present the different studies that have addressed this pivotal question.

In 1998 Jojovic *et al*. were the first to suggest that EMT leads to a transient loss of EpCAM expression during the migratory and early pro-migratory period by evaluating the expression at EpCAM in immunohistochemistry on breast and lung cell lines implanted in immunocompromised mice [84]. Then, gene expression analyses demonstrate that EpCAM is decreased in mesenchymal-like cancers. In breast cancer, EpCAM was down-regulated in mesenchymal lines relative to the epithelial cell lines [85] and in EMT-induced breast cancer cells [86, 87]. EpCAM was also one of the downregulated genes in an EMT gene signature developed from NSCLC cell lines [88]. In addition, a negative correlation between the activity of EMT-associated transcription factors SNAI1 [89], Slug [90] and ZEB1 [31] and EpCAM expression has been reported. Nevertheless, a direct functional link between EMT and EpCAM expression was missing. To directly investigate the impact of EMT on EpCAM expression, normal epithelial and various epithelial cancer cell lines were treated *in vitro* with transforming growth factor-β1 (TGFβ1) and tumor necrosis factor-α (TNFα), a combination that is known to induce EMT [91]. Following 72 h of cytokine treatment, immunofluorescence staining of cells showed decreased expression of the epithelial markers EpCAM and E-cadherin and increased expression of the mesenchymal marker vimentin compared with control cells. Moreover, growth factor stimulation leading to ERK2 activation (a key regulator of EMT) suppressed EpCAM expression. Similar downregulation of EpCAM at mRNA and protein levels was obtained in lung and esophageal cell lines after treatment with TGF [92]. More specifically, ERK2 suppresses EpCAM transcription through activation of EMT-associated transcription factors SNAI1, SNAI2, TWIST1 and ZEB1, which bind to E-box sites in the EpCAM promoter [91]. These results are in line with previous evidence that ZEB1 directly binds to the *EPCAM* promoter, leading to a ZEB1-dependent repression of *EPCAM* expression in human pancreatic and breast cancer cell lines [93].

**Figure 4:**
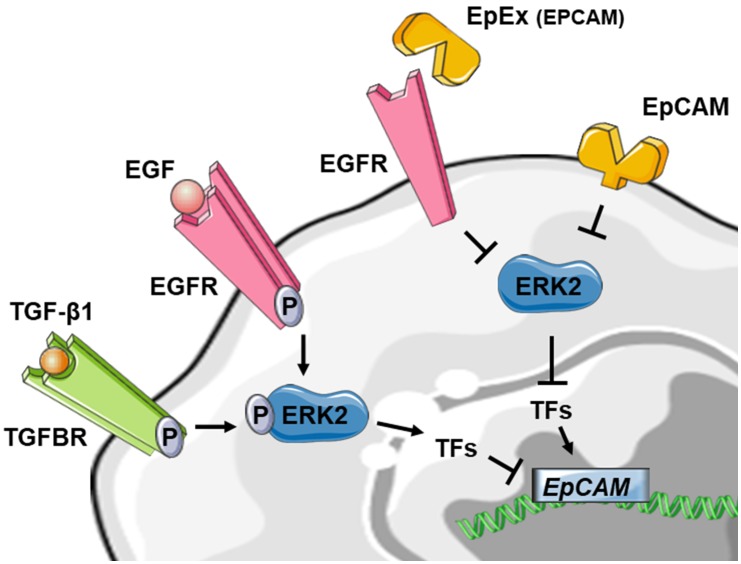
FIGURE 4: Reduced schematic of EpCAM regulation in the context of EMT. Stimulation of tyrosine kinase receptors via TGF-β1 or EGF is leading to ERK2 activation and suppresses *EPCAM* mRNA expression indirectly through activation of EMT-associated transcription factors (TF) like SNAI1, SNAI2, TWIST1 and ZEB1. The EpCAM protein on the other hand could also contribute to the regulation of EMT by suppressing ERK2 activity. The soluble EpEX-fragment is acting as an EGF competitor for EGFR, counteracted EMT via inhibiting of the EMTTF (Snail, ZEB1, and Slug) which leads to activation of *EPCAM* expression. For detailed review, see references [42, 91, 92].

Taken together, these results are in favor of a downregulation of EpCAM expression during EMT. Interestingly, EpCAM could also contribute to the regulation of EMT by suppressing ERK activity and SNAIL2 expression, defining a double-negative feedback loop (see above) [91]. As several double-negative feedback loops have been described in the regulation of EMT, notably feedback loops involving miR let-7 and LIN28, miR15a/16-1 and AP4, miR-34 and SNAI1 and miR-200 and ZEB1 [83], this double-negative feedback loop for EpCAM may be particularly noteworthy in the regulation of EMT in epithelial cancers. Furthermore, soluble EpEX was demonstrated as a ligand that induces specific activation of classical EGFR signaling pathways. Since EGFR signaling pathways promote EMT-characteristic phenotypic changes, the authors addressed whether EpEX could modulate EGF-dependent EMT in HNSCC cell lines and showed that soluble EpEX-Fc, acting as an EGF competitor for EGFR, counteracted EMT via repression of the EMTTF (Snail, ZEB1, and Slug) activation. This mechanism provides additional insights on the juxtacrine role of EpEx domain, shed in the tumor microenvironment after RIP of EpCAM (**Figure 4**) [42].

On the other hand, some studies have suggested a promoting role in EMT for EpCAM. Indeed, it has first been noticed that knockdown of EpCAM could inhibit the expression of EMT-transcription factors Snail and Slug in colon cancer [82] and that its overexpression could enhance TGF-β1-induced EMT in MCF-7 breast cancer cells [94]. More recently, in nasopharynx carcinoma, the mesenchymal markers N-cadherin, Vimentin, β-catenin and the EMTTF Slug were significantly upregulated, whereas the epithelial markers α-catenin and E-cadherin were decreased in the EpCAM expressing cells. Notably, this regulation involved the PTEN/AKT/mTOR pathway [45].

Therefore, regulation of EpCAM during EMT seems to be context dependent. As it is well established that cells during EMT no longer oscillate between full epithelial and full mesenchymal states but, rather, present high plasticity and can adopt a spectrum of intermediary phases [83]. Lineage tracing experiments in animal models to measure EpCAM expression in disseminating cells bearing different states in EMT transition would greatly help to confirm or infirm these results obtained in two dimentional cell culture experiments [83].

#### EpCAM role in invasion/migration

It can be, in a first sight, counter intuitive that a supposed adhesion molecule like EpCAM can promote the mobility of cells and tissues. However, implication of EpCAM in regulation of cell migration was studied during Xenopus gastrulation, a model to study morphogenetic movements [95]. EpCAM levels crucially regulate movements of cells in embryonic tissues via its EpIC domain acting as an inhibitor of a novel PKC isoform. Furthermore, in a conditional KO mouse model, loss of EpCAM impairs the migration of skin Langerhans cells [96]. On the opposite, migration of enterocytes in defective EpCAM mutant mice present significantly higher migration rates compared to wild type mice [14]. These data are noteworthy as acquisition of a migratory/invasive phenotype is also intrinsically linked with EMT. However, results on cancer cell lines about EpCAM are also conflicting and seem to be context dependent. Strong EpCAM overexpression was associated with enhanced invasion of breast cancer cell lines into extracellular matrix [60] and consisting results were observed upon silenced EpCAM expression due to the binding of the tumor suppressor p53 to promoter elements of the *EPCAM* gene [28]. In a subsequent study, the same team showed that the transcription factor activator protein 1 (AP-1) is an important downstream mediator of EpCAM signaling in breast cancer biology through the MEKK1-MKK7-JNK cascade [97]. Another way for EpCAM to promote invasion of breast cancer cells (ER negative) would be via upregulation of IL8 expression (a member of the CXC chemokine family associated with increased breast cancer invasion *in vitro)* but a precise molecular mechanism has not been identified yet [98]. However, specific ablation of EpCAM was also reported to increase invasion and migration in MCF-10A cells, underlying the importance of context dependence in our understanding of EpCAM signalization [91]. Direct involvement of EpCAM in the migratory phenotype of esophageal carcinoma was tested upon siRNA-mediated downregulation of EpCAM. An increased migration capacity of cells after EpCAM knock-down was paralleled by an increase of vimentin expression [92]. Moreover, in migrating esophageal carcinoma cells and in head and neck carcinoma cells, a progressive loss of EpCAM expression occurs at the membrane with the appearance of EpCAM positive speckles in the cytoplasm, suggesting EpCAM endocytosis and degradation [24, 92].

## RELEVANCE OF EpCAM AS A DIAGNOSTIC MARKER IN LIQUID BIOPSY ASSAYS

### EpCAM expression in CTC

Targeting EpCAM to capture CTC was a proven successful strategy by numerous clinical CTC studies on thousands of cancer patients demonstrating the prognostic relevance of EpCAM (+) CytoKeratins (+) CTCs in breast, prostate, lung or colorectal cancers, and other epithelial tumors, as reviewed elsewhere [99, 100]. Recently, the prognostic value of these cells in early stage breast cancer patients receiving neoadjuvant therapy was confirmed in a large meta-analysis [101]. Most of these clinical studies are based on the use of the CellSearch system that proceed with a ferromagnetic EpCAM-based capture of cells followed by their phenotypic characterization (cytokeratin (+), DAPI (+) CD45 (-)). This CTC assay has been extensively validated for its analytical accuracy and reproducibility [102]. Different EpCAM antibodies clones result in different CTC capture yield [103]. Thus, the question whether different antibodies binding different epitopes on the protein could improve recovery yield need further investigation [104].

However, the presence of EpCAM (-) CTCs in the blood circulation [105] has been also reported for patients with breast cancers [106, 107], colorectal cancer [108], or non small cell lung cancer [109]. Moreover, it was experimentally investigated in a mouse model that CTCs could escape from EpCAM-based detection due to EMT [110]. Together with the supposed necessity of EMT as a prerequisite for cancer dissemination, these results have raised doubt whether EpCAM-based capture is sufficient to escape all CTCs relevant to metastatic progression.

Therefore, many studies have focused their attention on CTCs with pronounced mesenchymal traits, expressing N-Cadherin, O-Cadherin, vimentin and fibronectin and some could find a correlation with clinical parameters like higher disease stage, presence of metastases, therapy response and worse outcome [111]. Nevertheless, there is no broadly used and established marker for specific enrichment of CTC with mesenchymal phenotype to date. Several approaches have been introduced, but none of them has been conclusively proven to enable a specific enrichment of cancer cells. Mesenchymal markers such as N-Cadherin and vimentin are frequently expressed on PBMCs (peripheral blood mononuclear cells). Therefore, these markers are not suitable for antigen-dependent enrichment of CTC and antigen-dependent enrichment techniques allowing a specific enrichment of CTC with mesenchymal phenotype have not been broadly implemented yet [112]. An alternative approach is to perform a negative selection for leukocyte markers in combination with the detection of mesenchymal markers [112].

To specifically capture CTCs with low or missing expression of EpCAM, additional epithelial specific cell surface markers, like EGFR, HER2, MUC1 [113, 114], have been applied to increase the sensitivity of CTC detection. Another option is the search for novel markers that are not downregulated by CTCs during their EMT and not expressed in blood cells. On the basis of comparative micro-array analyses, the *PLS3* gene was identified, which codes for the Plastin-3 protein, an actin-bundling protein known to inhibit cofilin-mediated depolymerization of actin fibers. Plastin-3 was demonstrated as a suitable new marker for CTCs in patients with colorectal cancer, especially in early-stage patients [115]. More recently, the recombinant VAR2CSA protein was found to bind a uniquely modified form of chondroitin sulfate, which is expressed by placental cells and cancer cells of both epithelial and mesenchymal origin. Its implementation in dedicated capture device led to a markedly enhanced CTC capture compared to EpCAM based method with the capacity to capture additional EpCAM negative CTC [116].

Another consequence of the drawbacks of EpCAM-based enrichment was the development of new label-free technologies for enrichment and detection of CTCs. These novel approaches are mainly based on the assumption that CTCs have different physical properties (size, deformability, density) than the surrounding blood cells. However, it can be assumed that these physical parameters might be also affected by EMT and EpCAM expression [87, 117].

Other studies have investigated the clinical relevance of cells that have not been captured by Cellsearch. These cells were captured from Cellsearch discard by filtration and identified with immunofluorescence staining against cytokeratin expression [118, 119]. Significant additional CTC numbers were identified with filtration in lung, breast and cancer patient samples. Clinical correlation with a worse outcome was found with EpCAM + CTC whereas EpCAM - CTC had no relation with overall survival. If the EpCAM - cells that have been collected are proven as tumorous, these results highlight the need to better discriminate the different populations of CTC regarding EpCAM expression.

Notably, concomitant detection of epithelial and mesenchymal markers has been reported within EpCAM+ CTC [120, 121]. Assessment of keratin expression as epithelial marker has also been successfully used in this context [122]. These results corroborate previous findings by Yu *et al*. demonstrating that a significant proportion of CTC bears a hybrid epithelial/mesenchymal phenotype [123] and are in line with the actual conception of EMT as a continuum, and not a binary switch between two extreme phenotypic stages [83]. It is also noteworthy here that the cells bearing the most mesenchymal phenotypes would not be the ones that present the highest metastatic capacity [83].

Taken together, despite the obvious shortcoming that EpCAM-based CTC detection might miss CTCs that have undergone EMT, EpCAM still outperforms other tested surface markers for CTC enrichment and detection, which is indicated by the fact that the CellSearch system still remains the only FDA-cleared technology for CTC detection. Better knowledge of the range of expression of EpCAM in CTC from patients and comparison of the clinical relevance of the different CTC populations with differential EpCAM expression could help to solve this ongoing debate.

### EpCAM in Extracellular Vesicles

Extracellular Vesicles (EVs) have emerged during the last ten years as critical mediators of cell-cell communication, involved in many normal physiological processes as well as in cancer progression. EVs can be isolated from bodily fluids including blood, urine, breast milk, ascites, bronchoalveolar lavage fluids and transport diverse nucleic acids (DNA, RNA, microRNA) and proteins as part of their function in intercellular communication [124]. Therefore, EVs might be interesting candidates as biomarkers for monitoring tumor evolution or response to therapy.

EVs is a general term to virtually describe any type of membrane particle released by any type of cell, into the extracellular space, regardless of differences in biogenesis and composition. Current criteria to distinguish between diverse EV populations are based on size, density, subcellular origin, function and molecular cargo. Based on a size, different categories can be distinguished: exosomes (30-100 nm diameter), microvesicles (MVs) (100-1000 nm diameter), and a more recently identified cancer-derived EV population termed “large oncosomes,” which are much larger than most EV types characterized to date (1-10 μm diameter) [125, 126].

EVs are highly enriched for tetraspanins, a protein superfamily that organize membrane microdomains termed tetraspanin-enriched microdomains (TEMs) by forming clusters and interacting with cholesterol and gangliosides and a large variety of TM and cytosolic signaling proteins [127]. Importantly, EpCAM has been associated to TEM via interaction with different tetraspanin proteins (CD9, CO-O29 as well as CD44 variant isoform) [128]. It has been recently demonstrated that EpCAM is essential for the gastrointestinal localization of some EVs secreted from the intestinal epithelia cells and implicated in the intestinal tract immune balance [129]. Interestingly, a proteomic analysis of the EV content from cancer cell lines, led Tauro *et al*. to define a distinct population of exosomes according to EpCAM expression [130] and showed colocalization of EpCAM with CD44 and claudin 7, proteins that are known to complex together to promote tumor progression [130]. In ovarian cancer patients, Im *et al*. detected the expression of EpCAM on exosomes from ascites at higher levels than in the control group of noncancerous ascites from cirrhosis patients [131]. More recently, EpCAM was also detected in exosomes fraction after surface protein exosomal profiling in plasma from pancreatic cancer patients [132]. Further investigations are necessary to confirm these data suggesting that EpCAM in circulation may represent a cancer-specific or at least cancer-associated exosomal biomarker.

Detection of soluble forms of EpCAM in blood serum of cancer patients has reported disappointing results, notably due to a lack of correlation of with important clinical end-points like overall survival in most studies [133-139]. Nevertheless, it might be interesting to compare the biological and clinical relevance of the exosomal forms of EpCAM with soluble EpEX domain.

## CONCLUSION

EpCAM expression is abundant in normal and malignant epithelial cells and the high immunogenicity of the EpCAM protein has enabled the development of specific antibodies that can be applied for immunohistochemistry analysis and enrichment of CTCs. EpCAM-based approaches for CTC detection have significantly contributed to validate the clinical relevance of CTCs in various cancer entities. Despite its widespread use as “epithelial marker”, the biological functions of EpCAM deserve more intense investigation. Besides affecting intercellular adhesion, EpCAM influences important other functions relevant to tumor progression including cell proliferation and cancer stemness, which suggests an active role of EpCAM in cancer metastasis.

Investigating the dynamic range of EpCAM expression on CTC in different cancer entities might provide new insights in the biology of cancer metastasis. A precise quantification of EpCAM expression in CTCs might help to better understand the prognostic role of the EpCAM+ CTCs demonstrated in numerous clinical studies. EpCAM and its signaling internal domain EpIC have been implicated in cancer stemness and the EMT process, suggesting that EpCAM is more than just a marker for CTCs. Understanding the regulation of EpCAM expression is important and in this context the methylation status of EpCAM in CTCs might provide important information. Besides CTCs, EVs isolated from the blood (and other body fluids) of cancer patients might provide complementary information. Thus, liquid biopsy analyses open new avenues to study the relevance of EpCAM for cancer metastasis and may lead to improvements in the personalized management of cancer patients.

## Abbreviations

aa: amino acid
CD: C-Domain
CTC: circulating tumor cell
CTE: congenital tufting enteropathy
EMT: Epithelial-Mesenchymal Transition
EpCAM: epithelial cell adhesion molecule
EV: extracellular vesicle
KO: kockout
RIP: Regulated Intramembrane Proteolysis
TEM: tetraspanin-enriched microdomains
TM: transmembrane
TY: Thyroglobulin type 1A domain
